# Rasch Analysis of the Disability Acceptance Scale for Individuals With Cerebral Palsy

**DOI:** 10.3389/fneur.2019.01260

**Published:** 2019-12-18

**Authors:** Eun-Young Park

**Affiliations:** Department of Secondary Special Education, Jeonju University, Jeonju, South Korea

**Keywords:** disability acceptance, cerebral palsy, Rasch analysis, psychometric, rehabilitation

## Abstract

**Background:** Acceptance of disability has been considered an important factor in rehabilitation procedures. The Disability Acceptance Scale (DAS) was used in a panel survey, and it is likely that this scale will be increasingly used. This study aimed to determine the psychometric characteristics of the DAS by applying a Rasch model, an application of item response theory.

**Methods:** Data were collected using the DAS with 84 individuals with cerebral palsy. The response data were analyzed for item fitness and item difficulty, rating scale fit, and reliability.

**Results:** Three of the nine DAS items had low fitness. Analysis of item difficulty showed that the item difficulty needs to be modified, suggesting the need to add some items with higher difficulty and some with lower difficulty. The 5-point Likert scale used in the evaluation questionnaires was not appropriate. An analysis of the six remaining items showed high levels of subject separation reliability and separation reliability of the items.

**Conclusions:** This study is significant because it identified the psychometric characteristics of the DAS through item response theory-based Rasch analysis and suggested the need to modify the item fitness and difficulty level. A modified six-item version of the DAS with a 4-point Likert scale was proposed as being more suitable.

## Introduction

Psychological problems associated with disability are associated with complications of rehabilitation, reduced quality of life (QOL), delayed recovery, and increased secondary medical problems (1). In addition, disability is often associated with negative emotions, such as sense of loss, hopelessness, and depression (2, 3). However, not all persons with disabilities experience psychological problems. Many people successfully adapt to dramatic changes in life, such as damage to one's body.

Researchers have suggested acceptance of disability as one element that explains why this diverse range of adaptations occurs (4). The concept of acceptance of disability is also used in terms of adaptation to disability (5). Disability acceptance is explained as being closely related to acceptance of loss (6) and is explained by changes in one's value system. Wright (7) described disability acceptance as a series of changes in values, in which an individual extends his or her range of values, places less importance on critical thinking about physical abilities, and places increased importance on one's remaining abilities. After refining Wright's (7) initial concept, acceptance of disability was redefined. The redefinition is a shift from comparing oneself with others to focusing on other assets or abilities one has, shifting one's focus from how one is damaged to other values, not interpreting damage as applying to all areas of one's life, and reducing the importance of one's body or appearance (7). Acceptance of disability has been explained from the perspective of looking at oneself as “other than ability” (8) and conceptualized as acceptance of loss. Furthermore, a person's acceptance of disability is likely to be associated with better adjustment to disability.

Positive acceptance of disability has traditionally been described as an important variable in rehabilitation (9) and a key factor in psychosocial adjustment (10). This is because positive acceptance allows people with disabilities to accept the reality of disability, to recognize their own values, and to continue their productive lives. This has led many researchers to emphasize acceptance of disability as a key factor of the rehabilitation process (11, 12). Recently, the life expectancy of individuals with cerebral palsy (CP) has increased, which is thought to be due to developments in medicine. Interest in the QOL of individuals with CP is increasing, and many studies have been conducted (13–15). A qualitative study to obtain the perspectives of adolescents with CP on factors influencing their QOL mentioned that personal characteristics, such as viewing problems as challenges to be overcome and acceptance of disability, were related to QOL (16). Disability acceptance in CP appears to be important for maintaining a good QOL. Both adolescents and parents indicated that good QOL was dependent on the adolescent accepting his or her disability (16).

As mentioned above, disability acceptance is a key factor in rehabilitation and psychosocial adjustment, and strategies are needed to improve disability acceptance for individuals with disabilities. This applies equally to individuals with CP. In South Korea, research on acceptance of disability has recently been actively conducted using the nine-item Disability Acceptance Scale (DAS) developed by Kaiser et al. (17). As panel data on acceptance of disability begin to be constructed, research on acceptance of disability is expected to become more prevalent in the future. Considering that acceptance of disability is an important variable in the rehabilitation process, future studies on the DAS used in the panel survey should be performed. Before an effective disability acceptance intervention program can be developed and its effectiveness verified, the reliability and validity of the measurement tool must be confirmed. However, no studies have examined the psychometric properties of the DAS for individuals with CP. When developing the DAS, Kaiser et al. (17) extracted nine items from 50 items developed by Linkowski (18). These items were specifically included to measure personal orientation toward disability.

Item response theory (IRT) differs from Classical Test Theory with respect to item property invariance. Item constancy invariance refers to item difficulty, whereas item discrimination means the characteristics of the item, and these are not changed by the characteristics of the participant. Evaluation of item property invariance is possible using IRT because IRT analysis uses an item characteristic curve with unique characteristics of each item. The Rasch model is the most frequently used method of IRT for evaluating the appropriateness of an item's suitability and item difficulty (19). Rasch analysis can analyze the difficulty and discrimination of each item. A strength of Rasch analysis is that the item characteristic estimation is not influenced by the characteristics of the target group (20, 21). Another strength of Rasch analysis is that it can be used to estimate the real ability of a participant based on the results of the analysis (22). Since the advent of IRT, studies that verify the psychometric properties of instruments have utilized IRT, and scales that have been previously standardized using Classical Test Theory have been revalidated using IRT (23). IRT can systematically and logically evaluate item relevance, as it assesses the completeness of the test and the need to remove or modify items more stringently than Classical Test Theory. For this study, the psychometric properties of the DAS for individuals with CP were verified using Rasch analysis based on IRT.

## Methods

### Data

To verify the psychometric properties of the DAS for individuals with CP, data from the Eighth Panel Survey of Employment for the Disabled (PSED) in Korea provided by the Korea Employment Agency for the Disabled were used. The survey was completed in 2016, and data from the secondary wave were used. The survey subjects were persons with disabilities who were registered under the Welfare Act for Disabled Persons in Korea and who were aged from 15 to 64 years old. The survey was conducted using Tablet PC-Assisted Personal Interviewing, in which a schedule of prior visits was recorded, and interviews were conducted at scheduled times.

### Sample

In the PSED, the list of persons registered with the Ministry of Health and Welfare was set as the population, and a two-phase sampling method was adopted. In this method, the number of extracted regions was adjusted and an appropriate number of samples for each type of disability, disability grade, and age were extracted. In a sample of the first-phase disability, a one-step colony extraction method was used to extract the regions, which were stratified based on type of disability, disability grade, and age. The stratification was extracted at a level that would satisfy the target error. Family members who knew details of their daily activities and economic activities were allowed to respond to the surveys on behalf of the individual with ID, in cases where it was difficult for the participants to respond directly to the surveys themselves. The panel included 4,577 respondents, and 84 individuals had CP.

Theoretically, the stability of an item calibration is its modeled standard error (SE). For a sample of N examinees, which is reasonably targeted at the items and that responds to the test as intended, average item *p*-values are in the range 0.5–0.87. Theoretically, the stability of an item calibration is its modeled SE, so that modeled item SEs are in the range 2/sqrt (N) < SE < 3/sqrt (N) (24). As a rule of thumb, at least eight correct responses and eight incorrect responses are needed for reasonable confidence that an item calibration is within 1 logit of a stable value. A two-tailed 99% confidence interval is ±2.6 SE wide. For a ±1 logit interval, this SE is ±1/2.6 logits. This gives a minimum sample in the range 4*(2.6)2 < N < 9*(2.6)2, that is, 27 < N < 61, depending on targeting. Thus, a sample of 50 well-targeted examinees is conservative for obtaining useful, stable estimates. Thirty examinees are enough for well-designed pilot studies (25). Eighty-four individuals with CP were a sufficient sample size for the analysis.

General characteristics of individuals with CP are shown in Table 1. Of 84 participants, 50 (59.5%) were male, and 34 (40.5%) were female. The majority of participants were aged from 15 to 29 (52.4%), and the next largest age group was 30–39 (26.2%). Regarding education level, 42 (50.0%) graduated from high school, 29 (34.5%) graduated above college, five (6.0%) graduated from middle school, and eight were (9.6%) below elementary school. Regarding marital status, 79 (89.3%) were unmarried, and nine were married (10.7%). Participants were predominantly unemployed (77.4%, *n* = 65). Nineteen participants were working (22.6%).

**Table 1:** General characteristics of the study subjects (*n* = 84).

**Category**	**Number**	**%**	**DAS mean**	**DAS SD**
**Gender**				
Male	50	59.5	3.19	0.68
Female	34	40.5	3.25	0.72
**Age**				
15–29	44	52.4	3.27	0.65
30–39	22	26.2	3.06	0.69
40–49	12	14.3	3.41	0.54
>50	6	7.1	3.03	1.19
**Education level**				
Below elementary school	8	9.6	3.08	1.02
Middle school graduate	5	6.0	3.16	0.62
High school graduate	42	50.0	3.08	0.69
Above college	29	34.5	3.48	0.61
**Marital status**				
Married	9	10.7	3.15	0.74
Non-married	75	89.3	3.20	0.66
**Employment**				
Yes	19	22.6	3.37	0.77
No	65	77.4	3.17	0.67

*DAS, disability acceptance scale; SD, standard deviation*.

### Measure

Among the 12 items for acceptance of disability used in this panel survey, nine items were derived from Kaiser et al. (17). The items had the following specific content: (1) “I feel satisfied with my abilities, and my disability doesn't bother me too much.” (2) “Although I am disabled, my life is full.” (3) “It makes me feel very bad to see all the things non-disabled people can do that I cannot.” (4) “My disability, in itself, affects me more than any other characteristic about me.” (5) “Because of my disability, I am unable to enjoy social relationships as much as I could if I were not disabled.” (6) “My disability causes me to think differently about everything.” (7) “How a person conducts him or herself in life is much more important than his/her physical appearance and ability.” (8) “Personal characteristics, such as honesty and willingness to work hard are much more important than physical ability.” (9) “There are many more important things in life than physical appearance.” The scale uses a 5-point Likert scale. Higher scores mean higher acceptance of disability level.

### Statistical Analysis

#### Item Fitness

The internal scale validity is also referred to as item fitness, which indicates how closely the actual item response matches the expected response from the Rasch model. The judgment is based on the infit mean square (MnSq) and outfit MnSq values. The infit MnSq values show an adverse reaction to the items at the competency level of the subject, and the outfit MnSq values show adverse reactions to items outside the competency level of the subject. The criterion for item fit is that when the infit and outfit MnSq are smaller than 0.5 or larger than 1.7, the item fitness is considered unacceptable (26). The closer to 1, the more fully the item reflects the construct being measured (27). An MnSq value >1.7 is called a misfit. This means that the item does not reflect the construct. When it is <0.5, it is called an unacceptable overfit (26). This means that there is a high likelihood that the item is redundant with other items. Misfitting or overfitting items should be reviewed and corrected or removed from scale.

#### Item Difficulties

The comparison between the individual attribute scores and the item difficulties was analyzed by using the distributions of the items and individuals with CP, which were included in one graph according to each respective attribute score to enable direct comparison. Because the individual attribute scores and the item difficulties were converted equally by using the logit scale, it was possible to have a direct comparison. When the ranges of two different distributions were consistent, that is, when the distribution ranges of the item difficulties were similar enough that the item difficulties could measure all ranges of the individual attribute scores and difficulties, the distribution was considered adequate (22).

#### Rating Scale

The rating scale function is the ability of the subject to understand the content of the categories and to distinguish between the characteristics of the categories correctly. It is also called response category appropriateness. The rating scale function is analyzed according to the following criteria. First, the category measure for each question should increase monotonically. Second, individual fit values for each rating over 1.5 with a 1.0 standard point suggest that the rating scale is not functioning effectively and the category should be merged afterward (28). If the response category does not increase monotonically, we will merge the categories to minimize this problem.

#### Separation Index

In Rasch analysis, the SE of measurement is calculated according to all proficiency levels apart from the sample group and is displayed in two varieties of the separation index. Person and item separation indexes are used for describing the reliability of the test in Rasch analysis. A larger separation index means that the measurement function level can be distinguished more strongly by the test. The criteria of the person separation index were as follows: (a) 1.50 represents an acceptable level of separation, (b) 2.00 represent a good level of separation, and (3) 3.00 represents an excellent level of separation (29).

## Results

### Fit of Items

Table 2 showed the item fit statistics according to entry order. There was one misfitting item (item number 7), which showed an infit MnSq value above 1.7. The results of fit indices are presented in Table 3 after removing misfitting or overfitting items. First, misfitting item number 7 was deleted. Then, item numbers 3 and 1 showed misfit values above 1.7. These items were deleted sequentially, and no misfitting or overfitting items were left.

**Table 2:** Item fit statistics according to entry order for nine items.

**Item content**	**Measure**	**SE**	**Infit**		**Outfit**	
			**MNSQ**	***Z*-value**	**MNSQ**	***Z*-value**
1. I feel satisfied with my abilities, and my disability doesn't bother me too much.	50.14	1.23	1.33	2.20	1.40	2.50
2. Though I am disabled, my life is full.	49.08	1.23	0.98	−0.10	1.00	0.10
3. It makes me feel very bad to see all the things non-disabled people can do which I cannot.	55.88	1.24	1.38	2.40	1.51	3.00
4. My disability, in itself, affects me more than any other characteristic about me.	51.50	1.23	0.58	−3.50	0.59	−3.20
5. Because of my disability, I am unable to enjoy social relationships as much as I could if I were not disabled.	49.84	1.23	0.51	−4.30	0.51	−4.10
6. My disability causes me to think differently about everything.	46.30	1.26	0.52	−4.00	0.52	−3.90
**7. How a person conducts himself in life is much more important than physical appearance and ability**.	**54.54**	**1.23**	**2.03**	**5.80**	**2.49**	**7.30**
8. Personal characteristics, such as honesty and willingness to work hard are much more important than physical ability.	45.35	1.27	0.86	−1.00	0.93	−0.40
9. There are many more important things in life than physical appearance.	47.70	1.25	0.76	−1.70	0.76	−1.80

*Measure, item difficulty, which refers to the difficulty of endorsing an item; MNSQ, mean square; SE, standard error; the possible range of MNSQ was from 0 to infinity; the expected MNSQ values for all items are 1.0. Bold, misfit item*.

**Table 3:** Item fit statistics according to entry order for eight, seven, and six items.

**Item No**.	**Eight items**						**Seven items**						**Six items**					
	**M**	**SE**	**Infit**		**Outfit**		**M**	**SE**	**Infit**		**Outfit**		**M**	**SE**	**Infit**		**Outfit**	
			**MNSQ**	***Z*-value**	**MNSQ**	***Z*-value**			**MNSQ**	***Z*-value**	**MNSQ**	***Z*-value**			**MNSQ**	***Z*-value**	**MNSQ**	***Z*-value**
1	50.08	1.35	1.72	4.22	1.90	4.80	**52.39**	**1.49**	**2.31**	**7.70**	**0.41**	**0.70**	**DELETED**					
2	49.59	1.35	1.02	0.20	1.03	0.30	50.84	1.49	1.07	0.40	0.70	0.70	51.74	1.78	1.21	1.30	0.78	0.79
**3**	**57.70**	**1.35**	**1.93**	**5.10**	**2.53**	**7.00**	**DELETED**											
4	52.48	1.34	0.60	−3.20	0.60	−3.10	54.37	1.48	0.74	−1.80	0.74	0.69	56.77	7.77	0.87	−0.90	0.79	0.78
5	50.50	1.35	0.53	−3.90	0.52	−3.80	51.95	1.49	0.59	−3.10	0.80	0.70	53.32	1.78	0.70	−2.20	0.84	0.78
6	46.24	1.38	0.47	−4.40	0.48	−4.20	46.75	1.52	0.43	−4.50	0.85	0.71	45.96	1.81	0.62	−2.70	0.86	0.80
**7**	**DELETED**																	
8	45.08	1.39	0.85	−1.00	0.89	−0.70	45.35	1.54	0.91	−0.30	0.74	0.71	43.98	1.82	1.24	1.70	0.76	0.80
9	47.56	1.37	0.80	−1.40	0.81	−1.30	48.36	1.51	0.89	−0.30	0.73	0.70	48.23	1.80	1.33	2.40	0.73	0.80

*Measure, item difficulty, which refers to the difficulty of endorsing an item; MNSQ, mean square; SE, standard error; the possible range of MNSQ was from 0 to infinity; the expected MNSQ values for all items are 1.0; Bold, misfit item*.

### Item Difficulty

**Figure 1:**
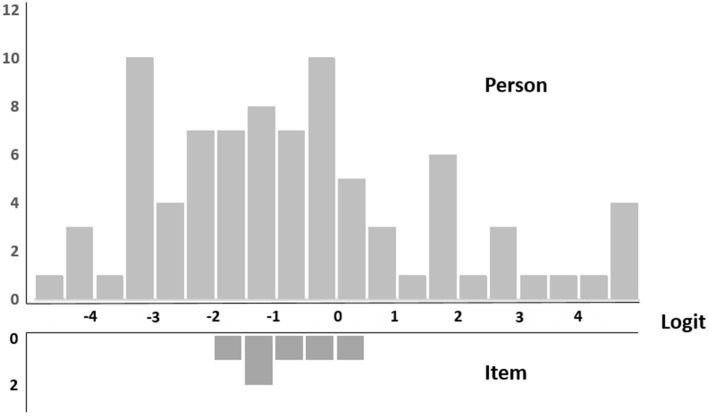
Item difficulty.

The map of individual proficiencies and item difficulties for the remaining six items of the DAS are presented in Figure 1. The most difficult item was number 4, and the easiest item was number 8. Twenty-six individuals with CP showed higher proficiency estimates than number 4, and 21 individuals with CP showed lower proficiency estimates than number 8.

### Appropriateness of the Rating Scale

The 5-point rating scale for the six items was compared with a 4-point rating scale for the six items, and the results are presented in Table 4. The results showed the appropriateness of the 4-point rating scale for six items. The fit statistics of each category level were below 1.5, and the analysis of the scale threshold showed that the threshold was proportional to the increase in scale scores in all subscales.

**Table 4:** Rating scale analysis of 5-point scale from 5-point six items and 4-point six items.

**Category level**	**5-point six items**						**4-point six items**					
	**Observed count**	**Observed count %**	**Observed average**	**Infit MNSQ**	**Outfit MNSQ**	**Structure calibration**	**Observed count**	**Observed count %**	**Observed average**	**Infit MNSQ**	**Outfit MNSQ**	**Structure calibration**
1	**15**	**3**	**−23.69**	**1.84**	**2.00**	**None**	**Collapse into 2**					
2	94	20	−13.23	0.82	0.82	38.39	85	19	−24.11	1.07	1.14	None
3	163	35	2.80	0.80	0.78	−9.79	160	36	−10.58	0.87	0.87	−23.62
4	170	36	15.64	0.90	0.92	8.57	170	39	3.01	0.94	0.98	−4.39
5	26	6	24.69	1.32	1.31	39.61	26	6	15.12	1.13	1.07	28.01

*MNSQ, mean square; Observed Count, the count of occurrences of this category; the expected MNSQ values for all categories are 1.0; Structure calibration is a calibrated measure of the transition from category below this category, so the value of Category Level 1 cannot be calculated and is marked as none. Bold, misfit item*.

### Separation Reliability

For the entire nine items, the person separation reliability value was 0.66, the person separation index was 1.41, the item separation reliability value was 0.84, and the item separation index was 2.25. For six items, the person separation reliability value was 0.86, the person separation index was 2.47, the item separation reliability value was 0.81, and the item separation index was 2.07 (Table 5). The separation index for persons and the six items of the modified DAS were identified as being acceptable.

**Table 5:** Person and item separation index of nine items and six items.

**Category**	**Person**		**Item**	
	**Separation index**	**Reliability**	**Separation index**	**Reliability**
9 items	1.41	0.66	2.25	0.86
6 items	2.47	0.86	2.07	0.81

### Level of Disability Acceptance

The descriptive statistics of the DAS are provided in Table 1. Females showed higher levels of disability acceptance than males. Individuals aged 40–49 with CP showed the highest levels of disability acceptance. Individuals with CP who have an above college education level showed the highest levels of disability acceptance. Higher levels of disability acceptance in non-married and employed individuals with CP were found.

## Discussion

The purpose of this study was to evaluate the psychometric properties of the DAS for individuals with CP using a Rasch model. The Rasch model, based on IRT, is widely used in the field of measurement and evaluation of education and medicine because it is a convenient way of analyzing data among various models, and is applied to various evaluation tools, as well as being used to supplement the shortcomings of existing evaluation tools (30).

In Rasch analysis, the infit MnSq index and the outfit MnSq index are standardized as indicators verifying the unidimensional structure of a measure. In general, the infit MNSQ index is a more sensitive index for responding to items close to the participants' ability level, and the outfit MNSQ index is a more sensitive index for responses to items far from the participants' ability level. Therefore, it is possible to prove linearity through the fitness index for the participants and items. If unidimensionality is confirmed, it proves that the scale is a valid measure of what one wants to measure (26).

The analysis of item fitness showed that item number 7, “How a person conducts him or herself in life is much more important than his/her physical appearance and ability,” was a misfitting item. Analysis of the remaining eight items showed that item number 3, “It makes me feel very bad to see all the things non-disabled people can do which I cannot,” was not suitable. Among the remaining seven items, item number 1, “I feel satisfied with my abilities, and my disability doesn't bother me too much,” had poor fitness. The remaining six items showed an appropriate fitness index.

The results of the item difficulty evaluation showed that the range of the difficulty level of the acceptance items was different from the participants' range of competence. To evaluate the disability acceptance of persons with CP, it is necessary to develop and add items with high as well as low difficulty. As shown in Figure 1 and Table 2, the range of the respondents' locations was wider than the range of difficulty levels of the items. The range of item difficulty was from 43.98 (Item 8) to 56.77 (Item 4). Additionally, the number of participants with high ability scores (above the most difficult item, namely, Item 4) was 19 (21.8%), indicating that the items were difficult for individuals with CP. The number of participants with low ability scores (above the least difficult item, namely, Item 4) was 26 (31.0%), indicating that the items were not difficult for individuals with CP. However, the appropriateness of the scale may vary depending on the specific characteristics of the respondent. Future research should investigate whether the difficulty of the DAS is also appropriate for younger people with ID, considering that this study collected data on participants who were at least 15 years old.

Analysis of the appropriateness of the rating scale showed that the 5-point Likert scale was not suitable, the response rate to the scale's 1-point was low, and the fitness score was not good. The 1-point response was deleted, and the measure was changed to a 4-point Likert scale. The results of the analysis showed that the 4-point rating scale was appropriate in that the fit indices for each response category were below 1.5 (22). The range of infit MnSq according to category level was from 0.87 (category level 3) to 1.13 (category level 5), and outfit MnSq range was from 0.87 (category level 3) to 1.14 (category level 4), indicating that the conditions for appropriateness were satisfied by the DAS and the 4-point scale reflected the characteristics of the item responses.

The separation index indicates how consistently the participants respond to the DAS items (31). The separation index is a measure of the standard deviation of the mean. The item separation index indicates how well the differences in each item are defined within the test. The person separation index indicates how effectively the test identifies and separates the participant's performance on the DAS. The larger the index, the more accurately the function level one is attempting to measure. In other words, the difference between items or subjects is well-defined or independent. Analysis of the separation index of the nine original DAS items showed that the person separation index was 1.41, the separation reliability index was 0.66, the item separation index was 2.25, and the separation reliability was 0.86. Analysis of the separation index of the revised six-item DAS showed that the person separation index was 2.47, the separation reliability index was 0.86, the item separation index was 2.07, and the separation reliability was 0.81. With a reliability coefficient of 0.70, the separation index of 1.5 was acceptable, the separation reliability factor of 80 was good, the separation index of 2 was good, and the separation reliability factor of 0.90 and the separation index of 3 were considered to be excellent. Therefore, the DAS consisting of six items can be judged to be acceptable and reliable.

It is clear that disability is a threat to the individual, but research suggests that this experience does not always have a negative impact. In other words, although disability negatively affects individuals for a certain amount of time, with the necessary individual characteristics and social and environmental resources in play, disabled persons can objectively evaluate their situation and regain a positive attitude to their lives (5, 31–34). Bretscher et al. (33) analyzed the satisfaction with life of disabled people and found that those who have adapted to disability evaluate their lives positively. The effect of acceptance of disability on life satisfaction has also been found for individuals with CP (16). Considering the importance of the psychological factor of acceptance of disability in rehabilitation (11, 12), the accurate measurement of acceptance of disability is also important.

This study is meaningful in that it confirms the psychometric properties of the DAS in individuals with CP. However, there are also some limitations. First, this panel survey only sampled people with disabilities who were aged 15 and above. Therefore, there is a limit to the extent to which the findings can be generalized to individuals with CP aged under 15. A second limitation also arises from the nature of the sample. Although we analyzed a large survey sample by using data from the PSED, this limits the representativeness of the sample regarding the population of individuals with CP.

In South Korea, the DAS has recently been actively used as part of a panel of data of the PSED. As the panel of data on acceptance of disability begins to be constructed, research on acceptance of disability is expected to become more active in the future. Considering that acceptance of disability is an important variable in services and programs, studies that examine the psychometric properties of the DAS used in the panel survey should be performed. It is undeniable that studies should use reliable and valid measurement tools, and any shortcomings should be identified. In this study, we attempted to confirm the suitability of the items, the difficulty of the items, and the adequacy of the rating scale by using Rasch analysis. We identified three unsuitable items in the DAS. A modified version of the DAS using a 4-point Likert scale and composed of six items was found to be appropriate. The modified DAS scale for measuring acceptance of disability for individuals with CP might be recommended for further studies that aim to verify the effects of acceptance of disability.

## Data Availability Statement

The data sets used and/or analyzed during this study are available from the corresponding author on reasonable request.

## Ethics Statement

This study is based on a secondary analysis of previously collected and publicly available data and therefore an ethics approval was not required as per applicable institutional and national guidelines and regulations.

## Author Contributions

E-YP has made the substantial contribution in interpretation and analysis of data, and drafting the manuscript.

### Conflict of Interest

The author declares that the research was conducted in the absence of any commercial or financial relationships that could be construed as a potential conflict of interest.
